# Low Heart Rate Variability Predicts Stroke and Other Complications in the First Six Postoperative Months After a Hip Fracture Operation

**DOI:** 10.3389/fcvm.2021.640970

**Published:** 2021-03-22

**Authors:** Gernot Ernst, Leiv Otto Watne, Frede Frihagen, Torgeier Bruun Wyller, Andreas Dominik, Morten Rostrup

**Affiliations:** ^1^Department of Anesthesiology, Kongsberg Hospital, Kongsberg, Norway; ^2^Section of Cardiovascular and Renal Research, University of Oslo, Oslo, Norway; ^3^Institute of Basic Medical Sciences, Faculty of Medicine, University of Oslo, Oslo, Norway; ^4^Oslo Delirium Research Group, Department of Geriatric Medicine, Oslo University Hospital, Oslo, Norway; ^5^Institute of Clinical Medicine, University of Oslo, Oslo, Norway; ^6^Division of Orthopaedic Surgery, Oslo University Hospital, Oslo, Norway; ^7^Technische Hochschule Mittelhessen (THM) University of Applied Sciences, Kompetenzzentrum für Informationstechnologie (KITE), Giessen, Germany

**Keywords:** heart rate variability, stroke, hip fracture, prediction, pneumonia, myocardial infarction

## Abstract

**Background:** One-year mortality after hip fractures is underestimated and is reported as 25%. An improved risk stratifying could contribute to a better follow up of these patients. Heart Rate Variability (HRV) is an easy point-of-care investigation and is been used in cardiology, endocrinology, and perioperative care. This observational study intended to explore relevant associations between HRV parameters and 6-months mortality and morbidity after a hip fracture.

**Methods:** One hundred and sixty-five patients admitted to two hospitals were included, and short-time HRV measurements (5 min, and 10 min at the two hospitals, respectively) were obtained. Mortality data were gathered by means of the Norwegian central address register. Patients, close relatives of patients, and in some cases their general physicians or nursery home physicians were interviewed 6 months postoperatively regarding the incidence of pneumonia, cardiac events, or stroke.

**Results:** One and hundred fifty-seven (95.2%) patients were followed up after 6 months post-surgery. Twenty-one (13%) died during this period. Twenty patients (13%) developed pneumonia, eight (5 %) stroke, and four (2%) myocardial infarction. No HRV parameter was associated with 6-month general mortality. However, patients who developed stroke had significantly lower High Frequency Power (HF, *p* < 0.001) and lower Very Low Frequency Power (VLF, *p* = 0.003) at inclusion compared to patients without complications. Patients who developed pneumonia had at the inclusion lower root mean square of successive differences (RMSSD, *p* = 0.044). Patients with a history of coronary heart disease (*n* = 41) showed a mortality of 7%. Mortality in this group was associated with standard deviation of beat-to-beat intervals (SDNN, *p* = 0.006), Total Power (TP, *p* = 0.009), HF (*p* = 0.026), and Low Frequency Power (LF, *p* = 0.012). Beta-blocker intake was associated with lower heart rate, but not with differences in HRV parameters.

**Conclusion:** In this exploratory study, we present for the first-time significant associations between different preoperative HRV parameters and stroke, myocardial infarction, and pneumonia during a 6-month period after hip fracture. HRV might be a simple and effective tool to identify patients at risk that would warrant better follow-up.

## Background

Hip fractures occur frequently in older people, and the incidence is increasing (1). The normal therapeutic approach is surgery. This is associated with a significant proportion of complications both perioperatively and during the hospital stay until discharge (2). Moreover, a hip fracture may also have effects on many aspects of the patient's well-being after they are discharged from the hospital (3, 4). Long-term mortality after hip fractures has to a large extent been neglected for many years. Some studies report a 5-year mortality of 55–68% (compared to 12% in population-based controls adjusted for age and previous hospitalization for serious disease). The highest mortality is reported during the first 6 months (5–7). One-year mortality rates of hip fractures in Norway are 25% according to the Norwegian Hip Fracture Register (8). The fact that one third of all postoperative complications and 50% of postoperative mortality are due to cardiac events, underlines the importance of risk estimation (9). Besides cardiac events, the risk for stroke after operations might be increased up to six times in patients over 80 (10), and anesthesia and surgery by themselves might increase the odds ratio to 2.9 (11). Currently, no ideal risk estimation tool to predict long term mortality exists (9, 12). Ischemic stroke risk models have still only moderate predictive value in different patient groups (13). Thus, simple risk estimation tools to identify low-risk and high-risk groups are needed.

Short-term heart rate variability (HRV) is a simple point-of-care investigation. Patient's heart rhythm is evaluated by an ECG measurement over 5–10 min. The beat-to-beat variance measured by QRS distances shows variability in different frequency areas. This time series can be analyzed with several mostly simple algorithms which can be differentiated between time domain, frequency domain, fractal analysis, or measures of entropy (14). Since 1996, a general accepted standard procedure has been used which makes studies comparable (15). In *time domain* the standard recommends standard deviation of beat-to-beat intervals (SDNN), and root mean square of successive differences (rMSSD). In *frequency domain*, one determines the frequency bands Total Power (TP), Very Low Frequency (VLF), Low Frequency (LF), High Frequency (HF), and the ratio of LF/HF. After this standard was established, HRV has been investigated as risk estimator in cardiology, perioperative care, and diabetes, among others (16). Short-term heart rate variability has been tested perioperatively in unselected patients (17, 18) and patients at risk of coronary artery disease (19), but not in patients undergoing surgery after hip fractures during a long-time follow up.

This prospective observational study intended to explore relevant associations between linear and non-linear HRV parameters and mortality and morbidity 6 months after hip fracture surgery. We hypothesized that decreased heart rate variability parameters might be associated with 6-months mortality, and 6-months incidence of pneumonia, stroke and myocardial infarction. We anticipated that such associations would be especially prominent in patients with known coronary heart disease. Since Beta-blockers might influence HRV results (20, 21), we also planned to look specifically into possible effects of these drugs.

## Methods

Patients with hip fractures admitted to Oslo University Hospital (OUS) and Kongsberg Hospital between 2008 and 2013 and with sinus rhythm in ECG were eligible for inclusion in the study. The patients at OUS were at the same time participating in another randomized study investigating the effect of geriatric care on cognitive function (22). Exclusion criteria were technical problems to take a short-time ECG (e.g., due to delirium), patients with unstable circulation, patients with operations the last month before admission, neoplasms, high energy trauma, and patients with short life expectancy. Patients signed written informed consent. In Oslo, substitute decision-makers were allowed to consent if the patients were not capable, evaluated by the including physician. An ECG signal was obtained within the 1st day after admission before operation and digitalized. We used Biocom ECG recorders (Biocom 3000 in Kongsberg, Biocom 4000 in Oslo), equipped with dry silver/ silver chloride ECG electrodes being mounted on the index fingers of the right and the left hand, respectively. After a relaxing period of 5 min an ECG signal was recorded over 5 min (Oslo) or 10 min (Kongsberg).

Signal measurement and processing were done according to recommendations the Task Force of the European Society of Cardiology and the North American Society of Pacing and Electrophysiology (15), and the methods are described in detail in recent publications (16, 23). Briefly, we used a sampling rate of 1,024 Hz (>512 Hz recommended for 5 min recordings). All ECGs were visually inspected and manually edited. Ectopic beats and noisy events were removed, and mean values was interpolated based on preceding and successive beats. Patients with more than 10% of ectopic beats or noise events were also excluded at this stage. Linear parameters (time domain: SDNN, rMSSD; frequency domain: HF, LF, VLF, LF/HF) were calculated by a Heart Rhythm Scanner - Version 2.0 – (Biocom Technologies – U.S.A). The frequency domain calculations were conducted non-parametrically with Fast Fourier Transform.

The sample size was estimated according to reference values reported earlier (24). The calculation was based on mortality. Assuming a 6-months mortality between 3 and 8% according to Dahl (25), 150 patients were expected to be sufficient to test the hypothesis of a significant association between mortality and HRV.

Normal distribution of data was tested with the Kolmogorov-Smirnov test. To test univariate associations, the independent samples *T*-test was applied. The Chi-Square test or Fisher's exact test were used in case of nominal data, as appropriate. Statistical analyses were conducted by the Statistical Package for Social Sciences (SPSS), release 18.0.3 (September 2010). Values are given in mean +/− SEM.

Every person in Norway is identified by a unique number in the Central Personal Register. Deceased patients were identified by the Norwegian central address register which provides exact data for the time of death. In addition, patients, close relatives of patients and in some cases their general physicians or nursing home physicians were interviewed 6 months postoperatively in the Kongsberg group regarding pneumonia, cardiac events, and stroke. In the Oslo group patients, close relatives of patients and in some cases their general physicians or nursing home physicians were interviewed regarding pneumonia, cardiac events and stroke within the first 6 months postoperatively. In both groups the results of the interviews were cross-validated by the hospital journals and—if relevant—nursery home journals regarding new hospital admissions within 6 months after the operation date.

The study protocol was reviewed and approved of the Regional Committee for Medical and Health Research Ethics of Southern Norway (11.1.2008, S-07307b) and the Data Protection Officer of Oslo University Hospital.

## Results

One hundred sixty-five patients (123 females and 42 males, mean age 80, 9 ± 9.9 Std. dev.) were included. All HRV data were normally distributed. At admission, one in four patients had an established diagnosis of coronary heart disease, one in three patients had hypertension and nearly one fifth COPD. Other patient details (other illnesses, medication,) are reported in Tables 1, 2.

**Table 1:** Preoperatively identified illnesses according to the case notes (*n* = 165).

**Illness**	**Number**	**%**
Coronary heart disease	41	25
Hypertension	62	38
Diabetes (insulin)	7	4
Diabetes (tablets)	15	9
COPD	31	19

**Table 2:** Preoperative drug treatment (*n* = 165).

**Drug treatment**	**Number**	**%**
Beta blocking agents	47	29
AT2 antagonists	23	14
ACE inhibitors	27	16
Calcium channel blockers	22	13
Diuretics	37	22
Glucocorticoids	13	8
Lipid lowering drugs	37	22
Sedatives	25	15
Antidepressive drugs	35	21
Antiepileptic drugs	7	4

One hundred fifty-seven (95%) patients were followed up at 6 months (with a similar proportion of illnesses, medication, ECG abnormalities and blood sample results compared to the original group).

Patients taking beta-blockers had a slightly lower heart rate (76 vs. 80, *p* = 0.04). There were no significant associations between intake of beta-blockers and HRV parameters. Thus, we did not include beta blockers as a variable in the statistical models.

Twenty-one patients (13%) died during the study period of 6 months after operation. Twenty-one patients (13%) developed pneumonia, eight (5%) stroke, and four (3%) myocardial infarction. Before discharge from the hospital, four (2.4%) patients deceased, 14 (8.5%) developed pneumonia, two (1.2%) patients developed stroke, and three (1.8%) myocardial infarction.

**Figure 1:**
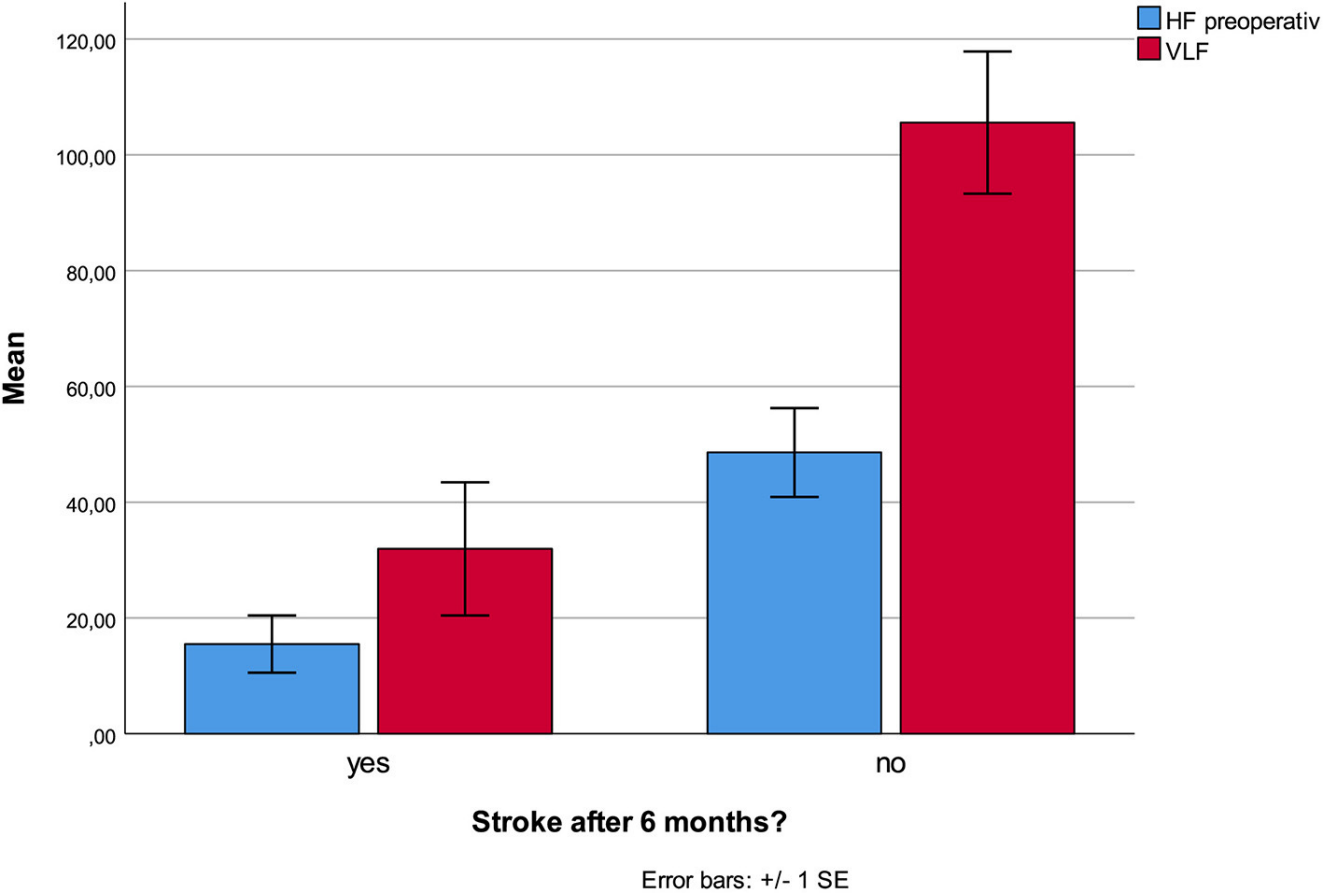
Lower HF and VLF at admission was associated with increased stroke incidence within the first six postoperative months.

**Figure 2:**
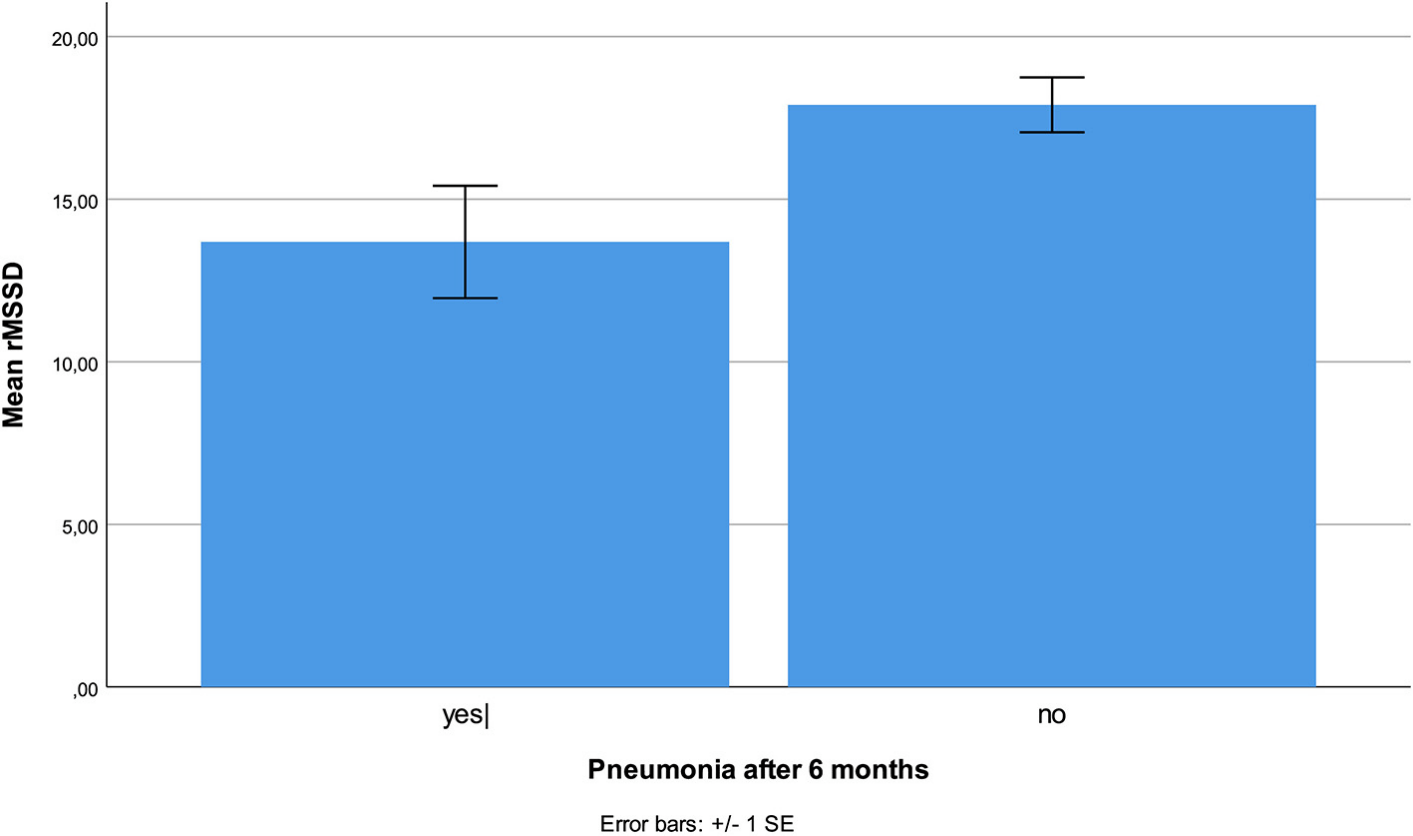
Reduced RMSSD was associated with higher incidence of pneumonia during the 6-month follow-up period.

**Figure 3:**
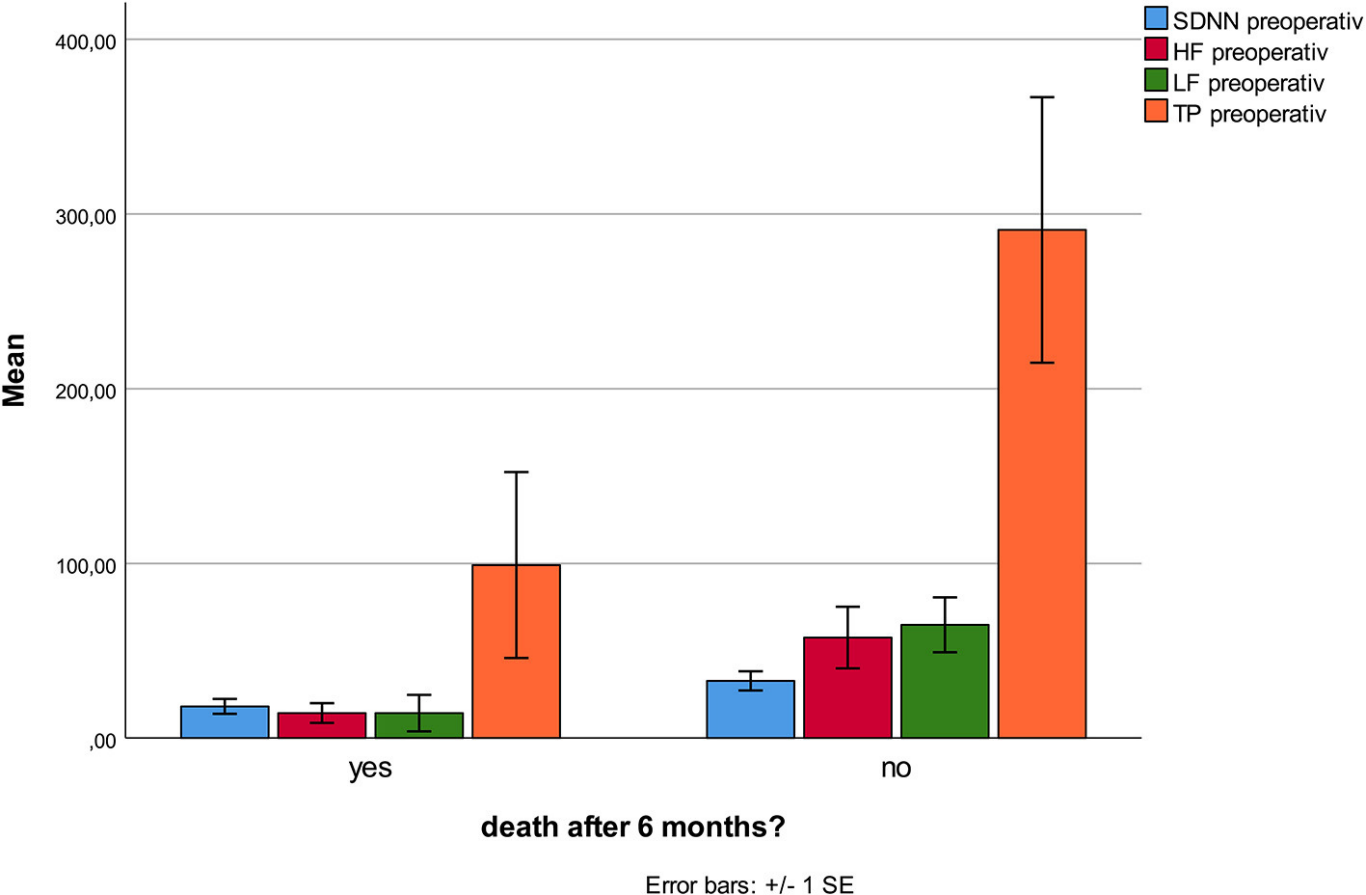
Mortality in the subgroup of patients with coronary heart disease is associated with lower TP, HF, LF, and SDNN.

No HRV parameter was associated with 6-month mortality. Patients who developed stroke had at inclusion lower HF (*p* < 0.001), and lower VLF (*p* = 0.003, Figure 1), compared patients without complications. There were no statistical differences regarding sex, age, or other prevalent illnesses at inclusion. Patients who developed pneumonia had at inclusion lower RMSSD (*p* = 0.044, Figure 2) compared to patients without complications. Patients developing pneumonia had significantly more often COPD (*p* = 0.004) and depression (*p* = 0.048). We also found that patients with coronary heart disease (*n* = 41) had a mortality of 7%. Mortality within this patient group was associated with SDNN (*p* = 0.006), TP (*p* = 0.009), HF (*p* = 0.026), and LF (*p* = 0.012, Figure 3). There were no statistical differences regarding sex, age, or other prevalent illnesses at inclusion in this group. All HRV results are presented in Table 3.

**Table 3:** HRV results in hip fracture patients without and with complications during the first 6 months postoperatively.

**Mean (S.E.M.)**	**No complications (*n* = 131)**	**Stroke (*n* = 8)**	**Pneumonia (*n* = 21)**	**Myocardial infarction (*n* = 4)**
Heart rate	78.1 (± 1.0)	84.0 (± 3.3)	78.8 (±2.6)	81.1 (±7)
SDNN (ms)	35.9 (± 2.67)	54.8 (± 20.3)	49.1 (± 10.4)	28.8(± 11.8)
rMSSD (ms)	17.5 (± 0.86)	14.7 (± 1.5)	13.7 (± 1.7)^*^	19.6 (± 6.4)
TP (ms^2^)	1515.6 (± 331.4)	2436.9 (± 1718.8)	2486.1 (± 1260.8)	263.7 (± 195.1)
LF(ms^2^)	164.1 (± 27.7)	101.3 (± 63.9)	131.6 (± 41.3)	56.3 (± 38.5)
HF(ms^2^)	54.4 (± 7.8)	14.6 (± 3.0)^**^	59.6 (± 21.8)	24.2 (± 15.7)
VLF (ms^2^)	101.9 (± 11.6)	31.9 (± 11.5)^***^	105.9 (± 36.4)	56.3 (± 38.5)
LF/HF	1.88 (± 0.18)	2.61 (± 1.06)	1.82 (± 0.30)	1.50 (± 1.00)

*Higher numbers indicate higher variability*.

* *p < 0.05*,

** *p < 0.005*,

*** *p < 0.001*,

*SDNN, standard deviation of all normal QRS-distances; rMSSD, square root of the mean squared differences of successive NN intervals; TP, Total Power; LF, Low Frequency Power; HF, High Frequency Power; VLF, Very Low Frequency Power*.

## Discussion

This prospective study showed strong associations between the incident of stroke and lower HF and VLF at time of admission. Pneumonia was associated with lower RMSSD. Mortality in patients with coronary heart disease was associated with lower SDNN, TP, LF, and HF. We found no association between HRV parameters and 6 months mortality.

Our study has some limitations. Due to practical circumstances, we recorded ECG for 10 min in Kongsberg, and 5 min in Oslo. Previous studies reported no relevant differences in HRV assessments of 5 or 10 min (26). Mortality was analyzed by retrieving data from the central address register and can be considered as very reliable. Our follow up percent was high (95%). Since the diagnosis of stroke, myocardial infarction and pneumonia was made at different places, different criteria might have been used. On the other hand, especially diagnosis of stroke and myocardial infarction are highly standardized, and most patients were treated for these illnesses in the same two hospitals. The diagnosis of pneumonia, however, was established in hospitals, nursing homes, or by general physicians. It is possible that we were not able to identify all patients with milder forms of pneumonia, either because they were not recognized as pneumonia or because some patients did not contact a physician. The HRV measurements were carried out according to international standards (15) which secures a sufficient quality. This is an exploratory study where we tried to identify relevant HRV parameters as predictors of mortality and morbidity. Many associations were tested. Thus, we should consider some associations with caution. However, the *p*-values of the associations between stroke and HF and VLF are very low, indicating a high probability. The association between pneumonia and rMSSD was in accordance with previous findings seen during the hospital stay of the patients (23).

This is the first study showing an association between HRV parameters and stroke incidence during a 6-month postoperative period. Of particular importance is that most strokes occurred after discharge of the hospital. Only two studies have reported HRV as a predictor of stroke previously, none of them in a perioperative setting. The Copenhagen Holter study followed 678 healthy persons between age 65 and 75 and calculated night-time SDNN between 2.00 and 2.15 a.m. based on a 15 min measurement. In contrast to 24 h SDNN and Mean NN, night-time SDNN was significantly associated with stroke in the follow-up period (27). In the Atherosclerosis Risk in Communities (ARIC) study, lower HRV was associated with higher risk of stroke, but only in participants with prevalent diabetes mellitus (28). HRV has else only been used to characterize patients after stroke (29).

Reduced HF is associated with reduced parasympathetic activity. Reduced parasympathetic activity has been associated with hypercoagulation and increased blood viscosity, and is possibly associated with arrhythmias (30). The VLF-component is a major determinant of physical activity and reflects possibly efferent sympathetic activity (31), in other sources modulated by the parasympathetic system (32), though its origin is controversial (33). Physiological studies indicate that VLF is mainly generated by stimulation of afferent sensory neurons in the heart provoking activation of feedback and feedforward loops (34, 35) and influenced by the renin-angiotensin system (32). Decreased VLF is often associated with increased inflammatory parameters like CRP, Il-6, and WBC (36). Increased inflammation and coagulation have often been associated with development of stroke (10, 37). Since both lower HF and VLF are also associated with these factors, they might reflect an increased tendency to inflammation and coagulation in patients predisposed to develop stroke. Most intervention studies have been conducted in cardiac surgery and showed that beta-blockers might prevent postoperative stroke in this patient group (38). Other predictive treatments, however, have shown conflicting results in studies (39). Our study is too small to establish HRV as a new risk assessment tool. A next step would be to include HRV-measurement together with other predictive parameters in a prediction model. This model and its possible guiding for interventions had to be tested in clinical investigations.

Increased incidence of pneumonia during the first 6 months of operation was associated with decreased rMSSD. A decreased rMSSD may indicate a lower parasympathetic activity and has been associated with immunologic changes in patients with hypertension (40), and in an experimental model where healthy human participants were treated with low dose endotoxin infusions (41). Decreased rMSSD has been suggested as an early marker of multiple organ dysfunction (42). The observed lower rMSSD in our patients with pneumonia might indicate an increased vulnerability to develop infections.

In patients with known coronary disease there was an association between mortality and lower SDNN, TP LF, and HF in our study. This is not surprising. SDNN, LF, and HF are all associated with increased risk for sudden cardiac death (43), although SDNN has been much more frequently measured in 24-h Holter monitoring. In risk populations with coronary heart disease these HRV parameters are correlated with long term mortality (44). Filipovic et al. followed patients scheduled for major elective non-cardiac surgery (e.g., vascular procedures of the abdominal aorta or lower limb, open intraperitoneal or intrathoracic procedures, major orthopedic procedures of the hip or spinal column, or major procedures on the neck). They observed an association between LF/HF <2 and mortality, but did not measure SDNN or TP (19). In our study, we did not find a significant lower LF/HF ratio, probably because LF and HF were reduced similarly. Our results confirm these earlier results.

We did not find associations between HRV parameters and general mortality. This is in contrast with other earlier studies. A recent met analysis including at all 21,988 participants without cardiac disease at baseline and followed up in cohort studies, demonstrated a robust association between decreased HRV and later cardiovascular events. Individuals with low HRV have about 40% increased risk of fatal or non-fatal CVD compared to individuals with high HRV. Recent studies had a follow-up of 9 and 15 years (36, 45, 46), as opposed to our study of only 6 months follow-up. However, we did find associations between HRV parameters, stroke, and pneumonia. Presence of stroke and recurrent pneumonia are associated with increased mortality (47, 48). Thus, we could expect a further increase in mortality in our study group after 6 months, secondary to events like stroke and pneumonia.

## Conclusions

We present for the first time a significant association between preoperative low HF, VLF, and stroke during a 6-month period after hip fracture. Pneumonia in this period was associated with low preoperative rMSSD. Mortality in cardiac patients was associated with low SDNN, TP, HF, and LF preoperatively. If these results can be confirmed, HRV might be a simple and effective tool to identify patients at risk. A better and more targeted follow-up of these patients might decrease morbidity and mortality.

## Data Availability Statement

The raw data supporting the conclusions of this article will be made available by the authors, on reasonable request.

## Ethics Statement

The studies involving human participants were reviewed and approved by Regional Committee for Medical and Health Research Ethics of Southern Norway (11.1.2008, S-07307b) and the Data Protection Officer of Oslo University Hospital. The patients/participants provided their written informed consent to participate in this study.

## Author Contributions

GE and MR developed the study protocol. GE, LW, and FF collected data. GE, LW, AD, and MR conducted the data analysis. All authors read and approved the final manuscript.

## Conflict of Interest

The authors declare that the research was conducted in the absence of any commercial or financial relationships that could be construed as a potential conflict of interest.
